# Efficacy of an mHealth Intervention (BRAVE) to Promote Mental Wellness for American Indian and Alaska Native Teenagers and Young Adults: Randomized Controlled Trial

**DOI:** 10.2196/26158

**Published:** 2021-09-15

**Authors:** Stephanie Craig Rushing, Allyson Kelley, Sheana Bull, David Stephens, Julia Wrobel, Joshva Silvasstar, Roger Peterson, Corey Begay, Thomas Ghost Dog, Celena McCray, Danica Love Brown, Morgan Thomas, Colbie Caughlan, Michelle Singer, Paige Smith, Kanku Sumbundu

**Affiliations:** 1 Northwest Portland Area Indian Health Board Portland, OR United States; 2 Allyson Kelley & Associates PLLC Sisters, OR United States; 3 mHealth Impact Lab Colorado School of Public Health University of Colorado aurora, CO United States

**Keywords:** American Indian, Alaska Native, adolescent, mental health, help-seeking skills, text messaging, mHealth, behavioral intervention, Indian health, mobile phone

## Abstract

**Background:**

Culturally relevant interventions are needed to help American Indian and Alaska Native (AI/AN) teenagers and young adults navigate common risky situations involving family and friends, including drug and alcohol misuse, dating violence, and suicidality. Leveraging *We R Native*, a multimedia health resource for Native teenagers and young adults, staff of the Northwest Portland Area Indian Health Board designed the BRAVE intervention for Native youth. The program is delivered via SMS text messaging and includes role model videos, mental wellness strategies, links to culturally relevant resources, and social support from family and friends.

**Objective:**

We aim to conduct a randomized controlled trial of the BRAVE intervention among AI/AN teenagers and young adults (aged 15-24 years) to assess its impact on their physical, mental, and spiritual health; their resilience and self-esteem; and their coping and help-seeking skills.

**Methods:**

From October to December 2019, we recruited 2334 AI/AN teenagers and young adults nationwide via social media channels and SMS text messages and enrolled 1044 participants. AI/AN teenagers and young adults enrolled in the study received either BRAVE SMS text messages, designed to improve mental health, help-seeking skills, and cultural resilience, or 8 weeks of science, technology, engineering, and math (STEM) SMS text messages, designed to elevate and reaffirm Native voices in STEM and medicine and then received the BRAVE SMS text messages. The impacts of the BRAVE intervention were tested using linear mixed-effect models and linear regressions.

**Results:**

A total of 833 AI/AN teenagers and young adults were included in the analysis. Individuals in the BRAVE and STEM arms showed significant positive trends over the course of the study for all outcomes, except cultural identity and help-seeking behavior. Mean scores were significantly different for health (*P*<.001), resilience (*P*<.001), negative coping (*P*=.03), positive coping (*P*<.001), self-efficacy (*P*=.02), and self-esteem (*P*<.001). Changes in help-seeking self-efficacy were significant in those exhibiting risky behaviors at baseline to exit (*P*=.01). Those who reported positive coping scores at baseline also reported better health on average; however, no difference was found in risky drug and alcohol use (*P*<.001). The number of participants who used SMS text messages to help themselves increased from 69.1% (427/618) at 3 months to 76% (381/501; *P*<.001) at 8 months. Similarly, the number of participants who used SMS text messages to help friends or family members increased from 22.4% (138/616) at 3 months to 54.6% (272/498) at 8 months.

**Conclusions:**

This is the first national randomized controlled trial of a mobile health intervention among AI/AN teenagers and young adults to test the efficacy of a mental wellness intervention in relation to STEM career messages. This study provides new insights for supporting the next generation of AI/AN changemakers.

**Trial Registration:**

ClinicalTrials.gov NCT04979481; https://clinicaltrials.gov/ct2/show/NCT04979481

## Introduction

### Background

There are 1.6 million youth aged ≤18 years in the United States who self-identify as American Indian and Alaska Native (AI/AN) [1]. With 573 federally recognized tribes throughout the United States, AI/AN populations are culturally diverse with distinct languages, cultural practices, ceremonies, traditions, and histories. Despite their immense cultural resilience and pride, AI/AN teenagers and young adults are disproportionally affected by stress, depression, and suicide [2]. As a population, AI/AN individuals experience substantial behavioral health needs that are compounded by limited access to mental health professionals and services [3]. Differences in risk behavior and health outcomes among AI/AN teenagers and young adults have been linked to poor economic and social conditions and historical trauma stemming from colonization [4]. Combined with chronic underfunding of the Indian Health Service, these conditions mean that many AI/AN teenagers and young adults do not have sufficient access to needed health services while living in medically underserved communities [3,5].

### Mobile Health Interventions

Mobile health (mHealth) technologies, including mobile devices, tablets, PDAs, and computers, are increasingly being used to address unmet health needs because they are cost-effective, developmentally responsive, and have the potential to reach large groups of AI/AN people living across the United States [6]. A recent analysis conducted by MarketCast found that AI/AN teenagers and young adults are open to social media about their mental health and coping strategies. From January to March 2020, participants mentioned mental health, stress, anxiety, and depression more than 1500 times on public social media channels [7]. A recent article by Around Him et al [8] underscored the relevance of social media outlets such as Twitter, Facebook, and Instagram. These platforms offer unique opportunities to address health disparities by building social connections, cultural advocacy, and supportive peer networks [8]. In recent surveys assessing the accessibility and acceptability of technologies to promote health, 93% of AI/AN youth reported having regular access to a smartphone and accessing the internet from their phones daily and 38% reported spending an average of 3-4 hours on social media per day [9].

SMS text messaging interventions are another example of how mHealth technologies are being used to fill the gaps in existing health delivery systems to improve health behavior. Mobile phones are nearly ubiquitous across age groups, socioeconomic classes, and language preferences. A meta-analysis of SMS text messaging to improve diverse health outcomes showed that interventions with tailored and personalized messages have greater effects than interventions without unique messages. However, questions remain about the best strategies for designing message content, the impact of generic versus more tailored SMS text messages, the optimal message timing and dose, and whether unidirectional or bidirectional messaging is important [10-13]. This study is the first to leverage SMS text messaging to promote mental health among AI/AN teenagers and young adults.

### Designing Interventions for AI/AN Teenagers and Young Adults

Culturally tailored and user-centered interventions are critically needed to increase the degree by which health messages are perceived as personally relevant by AI/AN youth, thus inspiring and supporting behavior change [6,14,15]. Although mHealth interventions (delivered via SMS text messaging and social media) have been used to improve multiple health outcomes [16], the extent to which mHealth can promote mental health for AI/AN teenagers and young adults is not yet known.

From 2015 to 2018, the Northwest Portland Area Indian Health Board (NPAIHB) carried out formative research involving youth as co-designers to create a behavioral intervention addressing alcohol misuse, intimate partner violence, and suicidality among AI/AN teenagers and young adults living across the United States. In 2015, we conducted key informant interviews with 10 Native young men to better understand their perceptions on alcohol misuse and the context of violence in their communities [17]. In 2016, we designed a series of theoretically informed, culturally relevant SMS text messages and a role model script to demonstrate and reinforce the skills described in the SMS text messages. In 2017, we pilot-tested the messages with Native youth and topical experts to assess the tone, content, and frequency of planned SMS text messages and video episodes.

Grounded in the principles of inclusion, equity, belonging, and diversity, the NPAIHB confirmed that the intervention could reflect the rich cultural diversity of tribes and urban youth living throughout the United States, and multiple phases of youth-driven research informed the design of the BRAVE intervention, the first mHealth intervention delivered via SMS text messages that uses culturally relevant images, narrative role model videos, and help-seeking resources [17].

This study has two aims: (1) to test the BRAVE intervention and (2) to test secondary associations, including self-efficacy, self-esteem, resilience, coping strategies, substance use, and cultural identity.

### Research Partners

#### Overview

The BRAVE intervention was designed and evaluated over 5 years through a series of community-based participatory research activities led by the NPAIHB (Multimedia Appendix 1). The first phase was carried out in collaboration with the Harvard School of Public Health and focused on better understanding alcohol misuse and the context of violence among Native males between the ages of 18 and 24 years using key informant interviews.

The second phase was carried out in collaboration with the NPAIHB’s *Tribal Health: Reaching out InVolves Everyone* project and *We R Native*. The NPAIHB is a regional, tribal nonprofit organization that represents 43 federally recognized tribes in Washington, Oregon, and Idaho. The Northwest Tribal Epidemiology Center is housed under NPAIHB and provides support through research, surveillance, and public health capacity building in partnership with the Northwest tribes. During the second phase of the study, the team designed and pilot-tested the intervention with AI/AN young men with a history of alcohol use and violence and 8 topical experts in alcohol prevention, alcohol treatment, violence prevention, health communication, and adolescent health.

During the efficacy phase of the study, the NPAIHB partnered with the mHealth Impact Lab at the Colorado School of Public Health. The lab works to facilitate the rapid and rigorous development, implementation, and evaluation of mobile and digital technology for health promotion and disease prevention to address inequalities in health outcomes. During this phase of the project, the NPAIHB recruited study participants and delivered SMS text messages, and mHealth led the design of data collection tools, data collection, and analysis. The partnership was supported by the Technology & Adolescent Mental Wellness (TAM) program, run by the Social Media and Adolescent Health Research Team and housed within the Department of Pediatrics at the University of Wisconsin-Madison.

#### We R Native

To reach AI/AN teenagers and young adults with culturally relevant health messages, the NPAIHB built *We R Native*—a holistic, multimedia health resource that reaches over 5000 viewers per day across its messaging channels. *We R Native* was designed using a youth-centered approach, in which youth were actively involved in selecting, writing, and designing their content. The service includes a website, an Ask Auntie Q&A service, an SMS text messaging service (text NATIVE to 97779), a YouTube channel, and social media accounts (ie, Facebook, Instagram, and Twitter). Given the widespread use of mobile phones by Native youth, mHealth interventions provide a promising tool to reach AI/AN youth who live across vast geographies in urban and rural communities.

*We R Native* regularly engages users to ensure that its tools and messages are relevant, timely, and culturally appropriate. In 2019, the team interviewed 13 AI/AN teenagers and young adults who regularly viewed *We R Native* channels (website, SMS text message, or social media). Participants shared ways in which *We R Native* messages had improved their mental health, cultural connectedness, sense of self-worth, and access to health resources, both for themselves and their loved ones. When asked to reflect on their own mental health concerns, participants mentioned grief and depression as the most common topics, followed by stress and mental wellness skills (eg, coping mechanisms).

These interviews also informed the final design of the BRAVE intervention, highlighting common coping strategies (ie, alcohol and drug misuse), preferred wellness strategies (ie, goal setting and self-care), help-seeking skills (ie, reaching out for help for themselves or a friend), and related protective factors, including cultural resilience, identity, and cultural pride [17]. Participants and key informants recommended expanding the inclusion criteria for BRAVE to females and expanding the age group to 15-24 years. Research is needed to test the efficacy of the BRAVE intervention using the expanded inclusion criteria.

#### Study Design and Objectives

To evaluate its efficacy, we conducted a randomized controlled trial (RCT) of the BRAVE intervention among AI/AN teenagers and young adults (15-24 years old) living across the United States and assessed its impact on their physical, mental, and spiritual health; their resilience and self-esteem; and their coping and help-seeking skills. Participants were surveyed at four time points (Multimedia Appendix 2 presents the primary and secondary hypotheses).

The use of RCTs in AI/AN communities is often considered an inappropriate study design, given its culture of inclusion that values shared benefits [18]. The NPAIHB used an attention control design to ensure that all participants could receive the same benefits from the study. An alternative study design offers superior methodological rigor compared with a no-intervention control arm [19]. An equivalent control arm was imperative to respect and honor the time and participation of the research participants. We selected science, technology, engineering, and math (STEM)–related SMS text messages because we felt that they would not interfere with the help-seeking skills demonstrated in the BRAVE arm and would be of equal value to participants and because we had access to STEM role model videos that could closely mirror the content and frequency in the study arm.

All data collection methods were approved by the Portland Area Indian Health Service Institutional Review Board in Portland, OR (principal investigator: SCR, *protocol no:* 1384639). A waiver for parental consent was requested and approved by the Institutional Review Board. In addition, a consent form was included on the cover page of the pre- and postsurveys. All instruments and data collection methods were reviewed and approved by the Portland Area Indian Health Service Institutional Review Board before data collection.

## Methods

### Participant Eligibility and Recruitment

The efficacy study included self-identified AI/AN teenagers and young adults aged 15-24 years. All participants were required to have a cell phone with SMS text messaging capabilities. Eligibility was described via SMS text messages and confirmed using a presurvey.

From September to December 2019, the NPAIHB recruited AI/AN teenagers and young adults via *We R Native* social media channels (ie, Facebook, SMS text message, and Instagram) [20]. Additional recruitment took place through listservs associated with tribes, Indian health and Indian education organizations, and human service organizations that serve AI/AN young adults (Indian Health Service, Methamphetamine Suicide Prevention Initiative, Healthy Native Youth, etc). Interested teenagers and young adults were asked to text the keyword BRAVE to 97779, which triggered a series of eligibility and consent SMS text messages. Over 2330 AI/AN teenagers and young adults texted BRAVE to learn more about the study.

### Enrollment and Randomization

To enroll in the study, participants were required to complete a presurvey. Those who met the eligibility criteria were randomized into the study (n=1044) using a 1:1 allocation ratio. mHealth used Excel (Microsoft) software to randomize participants into each study arm. There were a total of eight waves of enrollment from October to December 2019. Data were collected using web-based questionnaires in Qualtrics (SAP) at baseline and at 3, 5, and 8 months. In appreciation of their time, participants received a US $10 Amazon gift code for each survey they completed, up to US $40 per person.

### Intervention and Control Messages

AI/AN teenagers and young adults randomized into the intervention arm were immediately exposed to the BRAVE campaign, containing 8 weeks of SMS text messages with three messages per week. Participants in the control arm received messages designed to elevate and reaffirm Native voices in STEM and medicine. The STEM messages were delivered three times per week for 8 weeks. Both arms were designed to include a similar number of messages per week, with a combination of information, role model videos, images, and opportunities for reflection and engagement with the campaign (ie, reply for more information, provide a Q&A response, and click links to access resources). A paper by Stephens et al [20] described the theory that informed the design of campaign messages, as well as the sequence and content of the messages in greater detail.

### Data Collection

Data collection began on September 1, 2019. All four pre- and postsurveys were delivered using Qualtrics, an online data collection platform [21]. Questions collected information about standard demographic information, resilience measures, self-esteem, positive and negative coping skills, help-seeking self-efficacy, mental health, and cultural pride (see Table 1 and Multimedia Appendix 3 for the complete BRAVE survey). Data from the surveys were collected, stored, and maintained by mHealth Impact Lab.

**Figure 1:**
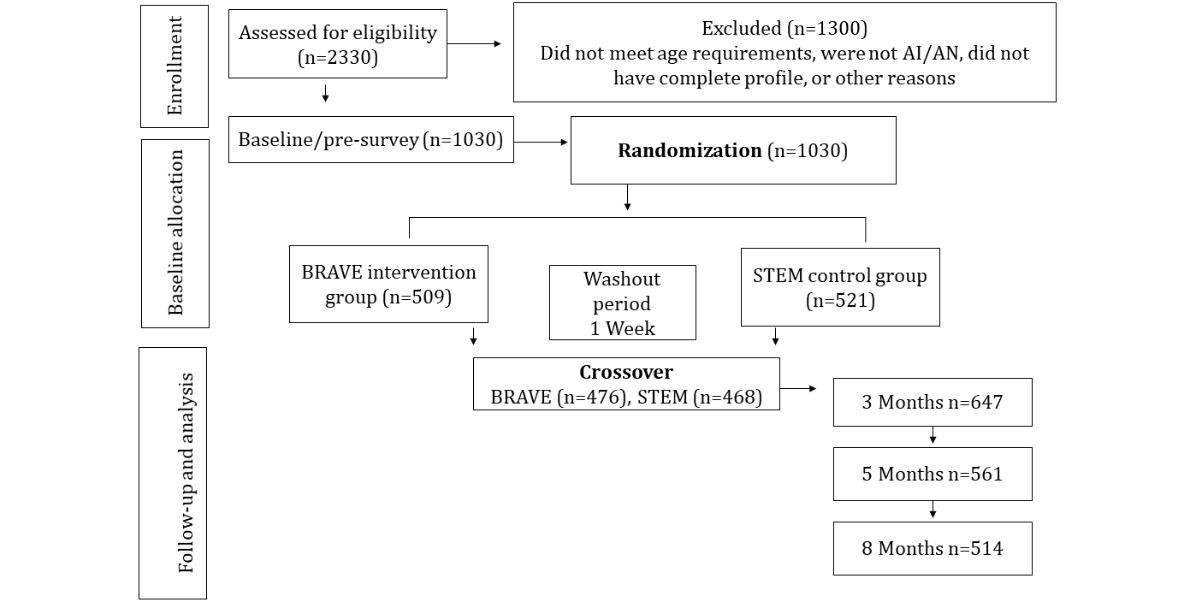
Participant recruitment and retention flow chart. AI/AN: American Indian and Alaska Native; STEM: science, technology, engineering, and math.

The retention was high across the two study arms. For BRAVE enrollees, only 41 participants opted out during the intervention, and only 25 opted out at crossover. For STEM enrollees, only 45 participants opted out during the control phase, and only 18 opted out at crossover. The washout period between the study arms was 1 week. In total, 86 participants opted out of the study during the first arm, and 43 opted out after the crossover, resulting in an 87% retention rate, as shown in Figure 1.

All participants were required to complete the baseline survey at the time of enrollment (presurvey). The presurvey was used to assess eligibility, randomize participants, and gauge baseline measures. Enrollees were asked to complete the same survey after the first set of messages (first postsurvey at 3 months). At the end of the intervention period, participants crossed over to receive the second set of messages and were asked to complete the survey a third time (second postsurvey at 5 months). The study team discontinued communication and asked participants to complete the final survey 90 days later (third postsurvey at 8 months), as shown in Textbox 1.

**Table 1:** Self-reported survey measures collected from BRAVE participants by survey dimension, sample question, analysis, and answer choices (N=35).

Survey dimension (number of items)	Sample question or statement and validated tool	Analysis	Answer choices
Health (3)	Rate your physical health, YRBS^a^ [22]	Changes in perceived physical, mental, and spiritual health	Excellent, very good, good, fair
Coping negative (2)	I’ve been using alcohol to make myself feel better, Youth Coping Responses Inventory [23]	Changes in problem drinking or drug use	Strongly agree, agree, disagree, strongly disagree, don’t know or not sure, don’t want to answer
Coping positive (5)	I’ve been taking action to try to make the unpleasant situation in my life better, Youth Coping Responses Inventory [23]	Proportion of respondents who report high scores of coping skills	Strongly agree, agree, disagree, strongly disagree, don’t know or not sure, don’t want to answer
Resilience (9)	I try to finish what I start, Child Youth Resilience Measure [24]	Proportion of respondents who report high scores of resilience	Strongly agree, agree, disagree, strongly disagree, don’t know or not sure, don’t want to answer
Self-efficacy (3)	I am able to reach out for help when I need it, Self-Efficacy Scale [25]	Proportion of respondents who report high scores of their own ability to succeed in different situations and tasks	Very confident, confident, somewhat confident, not at all confident, don’t know or not sure, don’t want to answer
Help-seeking skills (3)	I offer help or give advice to my friends who are struggling, Counseling Helping Questionnaire [26]	Proportion of respondents who report high scores of help-seeking	Often, sometimes, rarely, never, don’t know or not sure, don’t want to answer
Self-esteem (4)	As a whole, I am satisfied with myself, Rosenberg Self-Esteem Scale [27]	Proportion of respondents who report high scores of self-satisfaction, having good qualities, pride, self-worth, and self-respect	Strongly agree, agree, disagree, strongly disagree, don’t know or not sure, don’t want to answer
Cultural resilience and identity (6)	I enjoy my community’s traditions, Child Youth Resilience Measure [24]	Proportion of respondents who report high scores of connection, identity, and cultural pride	Strongly agree, agree, disagree, strongly disagree, don’t know or not sure, don’t want to answer

^a^YRBS: Youth Risk Behavior Survey.

**BRAVE data collection timeline and activities.**

**0-2 Weeks**

- Administration of survey 1 (baseline or presurvey) to determine eligibility, enrollment, and assignment to study arms

**2 Months**

- Sending out first set of messages (BRAVE intervention or science, technology, engineering, and math [STEM] control)

**1 Week**

- Administration of survey 2 (1st postsurvey)

**1 Week**

- Washout period

**2 Months**

- Crossover, second set of messages (BRAVE or STEM)

**1 Week**

- Administration of survey 3 (2nd postsurvey) and discontinued communication for 90 days

**3 Months**

- Administration of survey 4 (3rd postsurvey)

### Survey Measures

Survey measures were influenced by the Healing of the Canoe Survey (*Tribal Health: Reaching out InVolves Everyone project*) [28] and taken from validated survey tools, including the Youth Risk Behavior Surveillance Survey [22], the Youth Coping Responses Inventory [23], the Child and Youth Resilience [24], the Bandura’s Self-Efficacy Beliefs of Adolescents Scale [25], the Counseling and Help Seeking Questionnaire [26], and the Rosenberg Self-Esteem Scale [27]. The primary outcomes assessed were changes in AI/AN teenager and young adult intentions, behavior, and self-efficacy related to mental health, alcohol and drug misuse assessed by negative coping scales, and help-seeking skills. Outcomes were assessed using standardized scales and Likert-type ratings, wherein answers were converted to numeric scales, and averages and SDs were calculated for each outcome. For example, the health measure is an average score generated from three questions assessing physical, mental, and spiritual health. The secondary outcomes of the study included self-efficacy, self-esteem, resilience, coping strategies, substance use, and cultural identity. Table 1 outlines the survey constructs, the number of items for each construct, and examples for each question in the questionnaire (see Multimedia Appendix 3 for a complete list of questions).

### Data Analysis

The study data used in this analysis were collected by mHealth team members and managed using Qualtrics survey software [21] hosted at the University of Colorado Anschutz Medical Campus. All statistical analyses were performed using SAS statistical software (version 9.4) [29] and R (version 3.6) [30].

The study team explored the carryover effects between the BRAVE and STEM arms throughout the study period. Significant differences between the BRAVE and STEM arms were compared at baseline, crossover, and follow-up. Two analyses were conducted to explore the differences among primary outcome measures. First, a two-tailed paired *t* test was used to assess the treatment effect, comparing BRAVE with STEM arms using the difference in outcome scores for each measure. Second, a linear mixed-effect model (using a random effect for the subject) was used to test for changes over time. Treatment effects and changes in outcomes were considered significant at *P*<.05. The secondary outcome measures were analyzed using a series of statistical tests. Multiple linear regressions were used to assess the differences in the baseline and follow-up survey scores. Two models were used to assess the associations at baseline and changes in scores from baseline to study completion. A linear mixed-effect model (with random effects for subjects at 3, 5, and 8 months) was used to assess changes over time.

## Results

### Demographics

**Figure 2:**
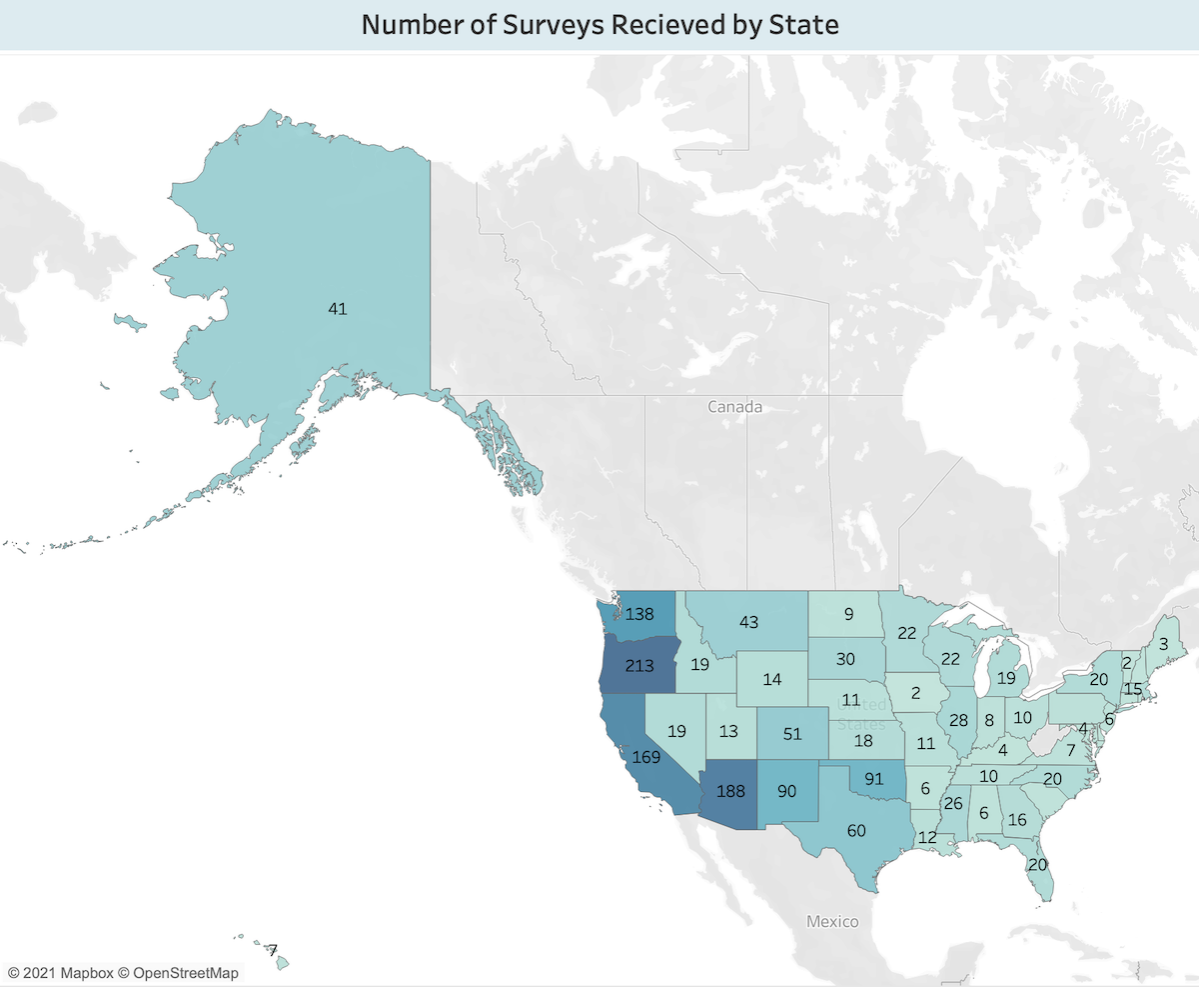
Map of the number of study participants by state in the United States.

Participants who completed the presurvey were aged 15-24 years and identified as AI/AN were included in this analysis (n=833; Table 2). Most participants were female (552/833, 66.3%). Three-fourth were straight or heterosexual (628/833, 75.4%). Most planned to go to college (440/833, 52.8%), although some were undecided (88/833, 10.6%). Participants most frequently identified as AI/AN only, followed by two and three races. Only 11.3% (94/833) of the participants identified as Hispanic. Study participants represented nearly every state in the United States. Most were from western states including Oregon (n=213), Arizona (n=188), California (n=169), and Washington (n=138) as shown in Figure 2.

**Table 2:** Summary of American Indian and Alaska Native teenagers and young adults who participated in the study at baseline, 3, 5, and 8 months (N=833).

Characteristic		Participant, n (%)
**Gender**		
	Female	552 (66.2)
	Gender fluid	9 (1.1)
	Male	241 (28.9)
	Transgender female to male	2 (0.2)
	Transgender male to female	1 (0.1)
	Two spirit	14 (2)
	Other (please specify)	6 (0.7)
	Prefer not to answer	8 (1)
**Sexual orientation^a^**		
	Asexual	6 (0.7)
	Bisexual	78 (9.4)
	Indigiqueer	7 (0.8)
	Lesbian, gay, or homosexual	42 (5.1)
	Pansexual	24 (2.9)
	Queer	7 (0.8)
	Straight or heterosexual	628 (75.8)
	Two spirit	12 (1.5)
	Other^b^ (please specify)	11 (1.3)
	Prefer not to answer	14 (1.7)
**Race**		
	Identify as AI/AN^c^ only	657 (78.9)
	Identify as 2 races	157 (18.9)
	Identify as s3	19 (2.3)
**Hispanic^d^**		
	Yes	94 (11.3)
	No	714 (85.8)
	Prefer not to answer	24 (2.9)

^a^Does not include four missing responses**.**

^b^Other responses were as follows: demisexual (n=2), questioning (n=2), straight (n=4), poly-panromantic asexual two spirit (n=1), no sexuality (n=1), and demisexual and biromantic (n=1).

^c^AI/AN: American Indian and Alaska Native.

^d^Does not include one missing response.

### Primary Outcomes

To test the study hypotheses and explore the primary and secondary outcomes of interest, the research team compared subjects with themselves and explored differences from pre- to postsurvey throughout the study period. Tests for differences between the two study arms did not show a significant difference within subjects, and statistical analyses revealed no carryover effects (Multimedia Appendix 4).

Participants in the BRAVE arm did not report better outcomes than those in the STEM arm. There were no significant differences in outcome measures at follow-up based on individual and aggregate survey measures. However, individuals in both study arms showed significant positive trends over the course of the study for all primary outcomes except cultural identity and help-seeking behavior.

### Longitudinal Effects

A review of 3-, 5-, and 8-month mean scores when controlling for treatment order showed a positive and upward trend for nearly all constructs measured (Table 3). Mean scores were significantly different for health (*P*<.001), resilience (*P*<.001), negative coping (*P*=.03), positive coping (*P*<.001), self-efficacy (*P*=.02), and self-esteem (*P*<.001; Table 3).

**Table 3:** Comparison of baseline and 3-, 5-, and 8-month mean scores among study participants (N=833).

Measure	Score, mean (SD)					*P* value
	Baseline	3 months	5 months	8 months		
Health	3.87 (0.88)	3.95 (0.87)	4.08 (0.85)	4.12 (0.83)	*<.001* ^a^	
Resilience	5.13 (0.56)	5.18 (0.52)	5.25 (0.51)	5.23 (0.49)	*<.001*	
Negative coping	2.88 (1.05)	2.89 (1.02)	2.92 (1.08)	2.92 (0.95)	*.03*	
Positive coping	5.04 (0.68)	5.13 (0.61)	5.17 (0.59)	5.22 (0.53)	*<.001*	
Self-efficacy	5.15 (0.61)	5.24 (0.59)	5.21 (0.6)	5.22 (0.55)	*.02*	
Self-esteem	5.07 (0.66)	5.17 (0.62)	5.18 (0.6)	5.19 (0.6)	*<.001*	
Cultural resilience and identity	5.4 (0.55)	5.38 (0.51)	5.4 (0.46)	5.4 (0.49)	.84	
Help-seeking skills	4.02 (0.89)	4.03 (0.85)	4.02 (0.81)	3.98 (0.8)	.06	

^a^Italicized values indicate statistical significance (*P*<.05).

### Health and Identity, Cultural Pride, and Resilience

Participants who reported better health at baseline reported stronger cultural identity, cultural resilience, identity, and cultural pride. Results from the linear regression (with the health score as the outcome and the cultural identity score as the covariate) showed an association between health and cultural identity (*P*<.001) at baseline. At baseline, health measures were positively associated with cultural identity*.* On average, those who reported better health also reported greater cultural resilience, identity, and cultural pride (*P*<.001). The *R^2^* value was 0.488, indicating that 48% of the variation in health scores was explained by cultural identity. Those who reported a positive change in health from baseline to 8 months were also more likely to report a positive change in cultural identity (Table 4).

**Table 4:** Results of the linear model and answers to secondary outcome questions.

Secondary outcome measures and covariates^a^			β		*P* value		Effect size
**Change in help-seeking self-efficacy**							
	Intercept	.452		*<*.001		0.021	
	Under 21	−.162		.18		—^b^	
	Gender male	−.154		.09		—	
	Risky baseline	−.280		.01		—	
**Health and cultural identity at baseline**							
	Intercept	1.23		—		0.092	
	Identity baseline	.488		*<*.001		—	
**Change in health and cultural identity**							
	Intercept	.144		*<*.001		0.027	
	Identity baseline difference	.241		*<*.001		—	
**Coping strategies, health, and substance use at baseline**							
	Intercept	0		*<*.001		0.153	
	Risky baseline	−.004		.94		—	
	Health baseline	.301		*<*.001		—	
**Change in coping strategies, health, and substance**							
	Intercept	.066		.02		0.028	
	Risky baseline	.114		.10		—	
	Health baseline	.123		.001		—	

^a^Each outcome is presented as a separate linear model. Effect sizes are measured by *R^2^*.

^b^The effect size is only reported for the intercept and compares the secondary outcome measures and covariates (eg, risky baseline and health baseline).

### Positive Coping Strategies

Participants who reported positive coping scores also reported better health on average, with no difference in drug and alcohol misuse (*P*<.001). The *R^2^* value was 0.301, indicating that 30% of the variation in coping was explained by better health. Those who reported a positive change in coping strategies from baseline to 8 months were also more likely to report a positive change in health during the study (*P*=.001). The *R^2^* value was 0.123, indicating that 12% of the variation in health was explained by positive coping strategies (Table 4).

### Help-Seeking Skills and Behavior

The changes observed in help-seeking self-efficacy were significant for those who reported risky behavior at baseline to 8 months (*P*=.01). The mean change from baseline in help-seeking behavior was −0.281 units lower for those exhibiting risky behavior at baseline. The *R^2^* value was 0.021, indicating that 2% of the variation in help-seeking self-efficacy was explained by the model containing only participants who reported risky behaviors at baseline. No differences were found among men or young adults aged ≥21 years (Table 4).

### User Engagement and Interaction

Participants reported and demonstrated a high level of interaction and engagement with the SMS text messages of the intervention. They reported reading the majority of the messages sent; this activity rate remained high throughout the study, with more than 91.2% (458/502) reporting all or most SMS text messages read at 8 months. The number of participants who shared SMS text messages with family and friends increased from 3 months (206/574, 35.8%) to 8 months (277/502, 55.2%; *P*<.001; Table 5).

**Table 5:** Changes in the use of help-seeking skills among study participants (N=833) at different timepoints.

Questions and responses^a^			Participants, n (%)						Trend			*P* value	
			3 months (n=647)		5 months (n=561)		8 months (n=514)						
**How many of the study’s SMS text messages did you read?**										Decreasing			*.01* ^b^
	Don’t want to answer	1 (0.1)		2 (0.3)		4 (0.8)							
	None	2 (0.3)		1 (0.2)		3 (0.6)							
	Some	35 (5.6)		43 (7.9)		33 (6.6)							
	Don’t know or not sure	6 (1)		4 (0.7)		4 (0.8)							
	Most	148 (24)		133 (24.5)		106 (21.1)							
	All	426 (68.9)		360 (66.3)		352 (70.1)							
**How many of the study’s videos did you watch?**										Decreasing			.60
	Don’t want to answer	3 (0.5)		2 (0.4)		2 (0.4)							
	None	57 (9.2)		38 (7)		30 (5.9)							
	Some	139 (22.5)		110 (20.2)		94 (18.7)							
	Don’t know or not sure	21 (3.4)		19 (3.5)		10 (1.9)							
	Most	143 (23.2)		163 (30)		224 (44.6)							
	All	254 (41.2)		211 (38.9)		142 (28.3)							
**How many of the messages did you share with your friends or family?**										Increasing			*<.001*
	Don’t want to answer	4 (0.7)		5 (0.9)		4 (0.8)							
	None	122 (19.8)		74 (13.6)		59 (12)							
	Some	254 (41.2)		204 (37.6)		144 (28.7)							
	Don’t know or not sure	30 (4.9)		18 (3.3)		18 (3.6)							
	Most	137 (22.2)		168 (31)		210 (41.8)							
	All	69 (1.2)		74 (13.6)		67 (13.4)							

^a^Results of the linear mixed-effect model with a random effort to subject (one for each question) based on 3-, 5-, and 8-month timepoints.

^b^Italicized values indicate statistical significance (*P*<.05).

### Changes in Help-Seeking Skills and Behavior

A comparison of 3-, 5-, and 8-month data showed significant improvements in help-seeking skills among BRAVE participants. Results from the linear mixed-effect model (with random effects for subjects) showed a significant increase. The number of participants who used the SMS text messages to help themselves increased from 69.1% (427/618) at 3 months to 76% (381/501) at 8 months (*P*<.001). Similarly, the number of participants who used the SMS text messages to help a friend or family member increased from 22.4% (138/616) at 3 months to 54.6% (272/498) at 8 months (*P*<.001; Table 6). At the end of the survey, participants were also asked to provide open-ended feedback on ways to improve the program for others. The team plans to conduct a qualitative analysis of user feedback to inform future improvements.

**Table 6:** User preferences and attitudes toward intervention observed among study participants at different timepoints.

Questions and responses^a^			Participants, n (%)							Trend			*P* value
			3 months (n=647)		5 months (n=561)		8 months (n=514)						
**How likely are you to use any of the resources you learned about if you, a family member, or friend need them?**										Decreasing			*.008* ^b^
	Don’t want to answer	3 (0.5)		2 (0.4)		3 (0.6)							
	Very unlikely	2 (0.3)		1 (0.2)		0 (0)							
	Unlikely	18 (3)		16 (3)		14 (3)							
	Don’t know or not sure	33 (5)		23 (4.2)		17 (3)							
	Likely	338 (54.8)		352 (64.8)		330 (65.7)							
	Very likely	223 (36.1)		149 (27.4)		138 (27.5)							
**Did you use any of the messages to help yourself?**										Increasing			*<.001*
	No	89 (14)		44 (8)		39 (8)							
	Did not need them	102 (16.5)		65 (12)		81 (16)							
	Yes	427 (69.1)		433 (79.9)		381 (76.1)							
**Did you use any of the messages to help a friend or family member?**										Increasing			*<.001*
	No	363 (58.9)		196 (36.1)		119 (23.9)							
	Did not need them	115 (18.7)		122 (22.5)		107 (21.5)							
	Yes	138 (22.4)		225 (41.4)		272 (54.6)							
**Did the messages encourage you to participate in any new wellness activities?**										Increasing			.07
	No	119 (19.3)		88 (16.3)		79 (15.8)							
	Yes	498 (80.7)		453 (83.7)		421 (84.2)							
**Is there anything else we can do to improve this program for others?**										Decreasing			*<.001*
	No	537 (87.6)		492 (90.7)		472 (94.8)							
	Yes	76 (12.4)		50 (9.2)		26 (5.2)							

^a^Results of the linear mixed-effect model with a random effort to subject (one for each question) based on 3-, 5-, and 8-month timepoints.

^b^Italicized values indicate statistical significance (*P*<.05).

## Discussion

### Principal Findings

This is the first national RCT of an mHealth intervention among AI/AN teenagers and young adults to rigorously test the efficacy of a mental wellness intervention in relation to STEM career messages, conducted in a 100% virtual format—recruiting, consenting, and surveying participants—via SMS text messaging (Multimedia Appendix 5).

Although our results did not fully support the proposed hypotheses, there were no measurable treatment effects between the two study arms. The findings indicate that both culturally relevant mHealth interventions improved health outcomes, an important programmatic finding. For both groups, most survey measures improved over time, including notable improvements in mental health, resilience, coping skills, and better self-esteem, despite the study conducted in the midst of the global pandemic. There were also significant reductions in reported alcohol and drug misuse by participants over the course of the study. Both study arms showed positive improvements in composite scores and trends, arguably the most promising outcome of all, demonstrating that culturally tailored SMS text messages can improve health and wellness outcomes for Native teenagers and young adults.

The lack of significant treatment effects may be partially explained by the lack of representation of Native youth and young adult experience in popular media, making the control arm similarly novel and inspiring to study participants. These findings have direct implications for future research. When designing RCTs for AI/AN groups, it is essential to consider what constitutes a control versus intervention arm. The study team believed that STEM messages would not interfere with the modeling of BRAVE-related skills. This RCT suggests, however, that the STEM role model videos had a similar effect on the study participants.

The need for culturally relevant STEM interventions is also critical for Native youth and young adults [31]. Native students are chronically underrepresented in training programs for STEM professions. Expanding the diversity of the STEM and medicine workforce is needed to meet the unique needs and worldviews of AI/AN communities. Messaging campaigns, such as those offered in the control arm, may inspire Native youth and young adults to join the STEM workforce pathway.

### Health and Identity, Cultural Pride, and Resilience

At baseline, physical, mental, and spiritual health measures were positively associated with cultural identity. This suggests that resilience, identity, and cultural pride may be associated with better health among AI/AN teenagers and young adults. These findings are consistent with a previous study that reported that AI/AN identity may be protective against a myriad of risk behaviors, including alcohol and drug misuse [32]. This research adds to a growing body of evidence that recognizes the foundational importance of building cultural pride and positive self-worth in adolescent health programs for AI/AN youth, further demonstrating that health and identity are inextricably intertwined.

### Positive Coping Strategies

Notably, participants who reported better coping strategies at baseline (ie, having healthy outlets to use when stressed, taking active steps to improve mental health, or using positive self-talk to overcome unpleasant feelings) also reported better health on average but did not have any difference in risky behavior compared with their peers. In addition, participants who reported a positive change in coping strategies over the course of the study were more likely to report a positive change in physical, mental, and spiritual health during that period. These findings reinforce a previous study on AI/AN populations, which suggested that having healthy coping strategies may directly impact health [33]. Health advocates can build on these findings by encouraging students to find and practice healthy coping strategies that reflect their unique skills and preferences.

### Alcohol and Drug Misuse May Compromise Help Seeking

Although we hypothesized that the BRAVE intervention might be more effective to the original audience of AI/AN males aged 21-24 years involved in formative research, it was not correct; we found that changes in help-seeking self-efficacy were not significantly different from younger AI/AN males aged 18-20 years. This was a welcome surprise, reinforcing our decision to expand the eligibility criteria for the study.

There was, however, one significant difference among males at baseline: males who reported greater alcohol and drug misuse also reported lower help-seeking behavior. Previous research in other populations suggests that help-seeking interventions are most effective when an individual is symptomatic and felt the need to ask for help [34]. Future research should determine the points or conditions in which help-seeking interventions, such as BRAVE, are the most beneficial for reducing alcohol and drug misuse.

### Help-Seeking Skills and Behavior

Finally, self-reported help-seeking skills (ie, ability to recognize when a friend is struggling, and willingness to contact a helpline, seek counseling, or treatment if needed, for themselves or a friend) were moderate to high among study participants. Healthy participants with high help-seeking characteristics who entered an intervention showed less change in these attributes over time.

Similarly, self-reported data showed a high level of interaction and engagement throughout the study; however, this engagement did not translate to a significant intervention effect on the primary outcomes of interest. Future work could explore the efficacy of the BRAVE intervention among AI/AN teenagers and young adults who have been identified as high-risk due to their involvement with juvenile justice systems, foster care, treatment, or behavioral health programs.

### Strengths and Limitations

The BRAVE study has several strengths and limitations. A major strength of the study was the RCT design, where random allocation allowed for a clean comparison between the intervention and control groups. The biggest strength of BRAVE was its development with input from AI/AN teenagers and young adults, tribal health educators, and topical experts. The focus of BRAVE on culturally relevant images, language, and the use of role model videos demonstrated respect for diversity and value. BRAVE created health communication messaging that AI/AN youth and young adults could identify with.

There are several limitations that should be noted. First, all data were self-reported. The sample was recruited largely through *We R Native*, which is a multimedia health and wellness resource. Participant familiarity with *We R Native* may have influenced their responses and participation. The initial goal was to enroll 125 participants from the 12 Indian Health Service geographic regions (n=1500) to explore the efficacy of BRAVE across regional differences. However, only 1030 participants, which is an insufficient sample size to explore regional differences, enrolled in BRAVE. Of these, only 833 were included in the analysis to improve data quality. The washout period of 1 week between the intervention and control arms may not have been sufficient to observe an effect in the treatment and control conditions. Finally, most participants reported high or favorable scores on the survey measures at baseline. This may have contributed to the lack of treatment effects between the two arms.

### Conclusions

This study builds upon the extensive digital research activities already carried out by the NPAIHB and the mHealth Impact Lab and fills a critical need for evidence-based interventions that reflect the unique needs and worldview of AI/AN teenagers and young adults. This research provides new information on the development of mHealth interventions for AI/AN youth using evidence-based strategies and furthers our understanding of the connections between health and cultural resilience, identity, and cultural pride. This work demonstrates the feasibility of recruiting a national sample of AI/AN teenagers and young adults online, which is largely due to the trusted nature of the *We R Native* platform. It also underscores the acceptability of SMS text messaging as a vehicle to promote and support mental health and wellness, addressing a priority concern among AI/AN teenagers and young adults.

Researchers, practitioners, policy makers, and AI/AN-serving organizations may use this example to improve the relevance, efficacy, and use of other mHealth interventions to reach high-risk, underserved populations. BRAVE lessons can be easily integrated into the flow of services provided by clinics, schools, treatment centers, and other community-based programs and can be tailored to the needs and time constraints of any setting [35]. Future research may address the recommendations offered by study participants to improve campaigns for future users and assess the uptake of the service outside the study setting. Native youth and young adults can now text BRAVE to 97779 or STEM to 97779 to receive the SMS text message sequence on their own.

Taken together, these results demonstrate the importance of culturally relevant health resources to support AI/AN teenagers and young adults as they navigate common risky situations involving family and friends, including alcohol and drug misuse, dating violence, and suicidality. User-designed mHealth interventions are critical to reach and engage at-risk populations and nurture lifelong decision-making skills. In the words of one participant, demonstrating the power and promise of BRAVE, “It encouraged me to better my health not only for myself but for the future generations.”

## Appendix

Multimedia Appendix 1 Healthy Native Youth BRAVE Website and Curriculum.

Multimedia Appendix 2 BRAVE study hypotheses.

Multimedia Appendix 3 BRAVE survey questions.

Multimedia Appendix 4 Treatment effect results and mean differences.

Multimedia Appendix 5 Graphical abstract of the BRAVE study.

Multimedia Appendix 6 CONSORT-EHEALTH checklist (V 1.6.1).

## Abbreviations

AI/AN: American Indian and Alaska Native
mHealth: mobile health
NPAIHB: Northwest Portland Area Indian Health Board
RCT: randomized controlled trial
STEM: science, technology, engineering, and math
TAM: Technology & Adolescent Mental Wellness
