# Supplementary material for: Efficacy of an mHealth Intervention (BRAVE) to Promote Mental Wellness for American Indian and Alaska Native Teenagers and Young Adults: Randomized Controlled Trial
Source: JMIR Ment Health. 2021 Sep 15;8(9):e26158. doi: 10.2196/26158 (PMC8482172; doi:10.2196/26158)
Supplement: Multimedia Appendix 1 [file mental_v8i9e26158_app1.pdf]

BRAVE

GIVE US FEEDBACK

COMPARE CURRICULA

ABOUT | TRAINING | LESSONS PLANS | SUPPORTING MATERIALS | CULTURAL RELEVANCE | EVALUATION | REFERENCES

BRAVE USER'S GUIDE

Updated:02/13/2021

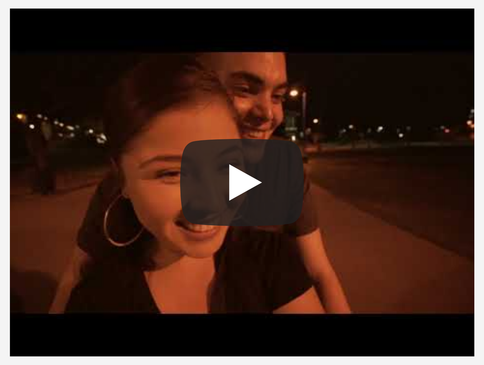

American Indian and Alaska Native young adults are strong and resilient. Interventions designed to improve their mental health and help-seeking skills are especially needed, particularly those that include culturally-relevant resources and relatable role models. The multi-media BRAVE intervention was designed for American Indian and Alaska Native teens and young adults to amplify and reinforce healthy social norms and cultural values, teach suicide warning signs, prepare youth to initiate difficult conversations with peers and trusted adults, encourage youth to access mental health resources (i.e. tribal clinics, chat lines), de-stigmatize mental health services, and connect youth to trusted adults.

The intervention can be flexibly delivered in three formats. It includes text messages, role model videos, a user's guide, and small group activities. The lessons can be easily integrated into the flow of services provided by clinics, schools, treatment centers, and other community-based programs. BRAVE can be implemented by a variety of support staff and can be tailored to the needs and time constraints of any setting.

AGE GROUP DESIGNED FOR: High School, Young Adults

LGBT INCLUSIVE: No

PROGRAM SETTING: Flexible

DURATION: 1 lesson at 30 minutes; 1 lesson at 45 minutes; or 7 lessons at 45 minutes apiece

COST TO PURCHASE: Free

TEACHER TRAINING OR CERTIFICATION REQUIRED: No

STUDENT TO TEACHER RATIO: Up to 75:1

EVIDENCE OF EFFECTIVENESS: Promising Practice

ENDORSEMENTS: N/A

PROGRAM CONTACT INFORMATION

Stephanie Craig Rushing
scraig@npaihb.org
https://www.wernative.org

GET STARTED

BACK TO ALL CURRICULA

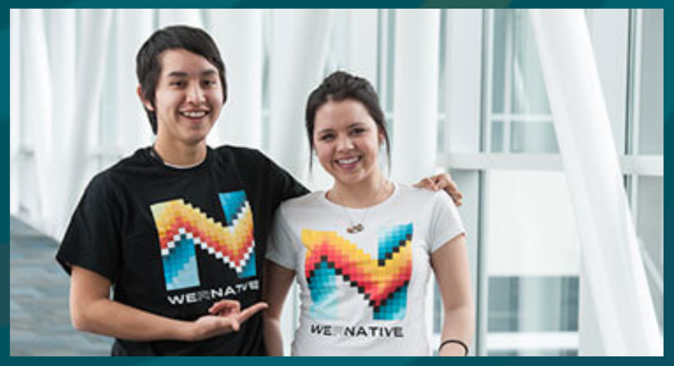

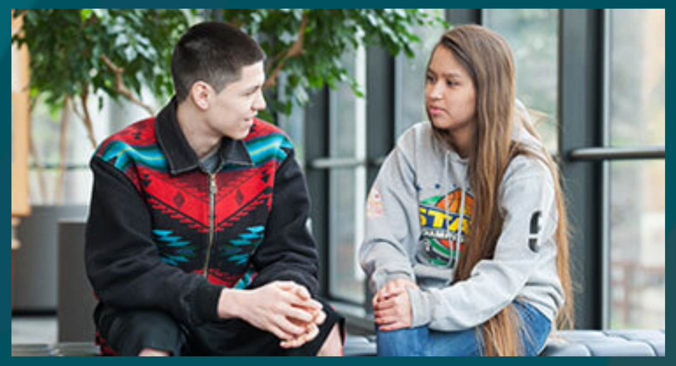

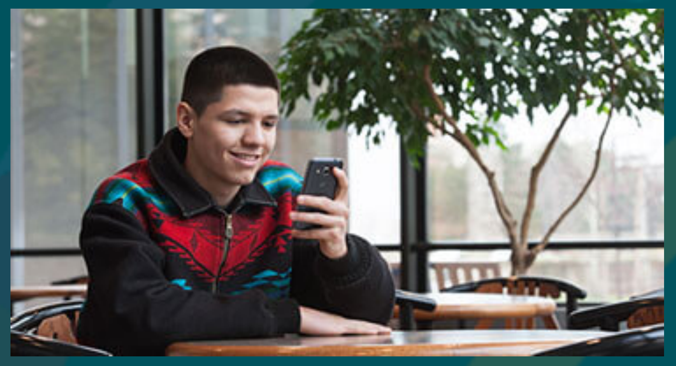

SIGN UP TO RECEIVE CURRICULA UPDATES

FIRST NAME | LAST NAME | TRIBE OR ORGANIZ/ | CELL PHONE | EMAIL ADDRESS

CAPTCHA

I'm not a robot

reCAPTCHA
Privacy - Terms

SIGN UP

© Copyright 2020 Northwest Portland Area Indian Health Board
