# Supplementary material for: Efficacy of an mHealth Intervention (BRAVE) to Promote Mental Wellness for American Indian and Alaska Native Teenagers and Young Adults: Randomized Controlled Trial
Source: JMIR Ment Health. 2021 Sep 15;8(9):e26158. doi: 10.2196/26158 (PMC8482172; doi:10.2196/26158)
Supplement: Multimedia Appendix 2 [file mental_v8i9e26158_app2.docx]

## Multimedia Appendix 2

The list below describes the BRAVE study hypotheses with primary and secondary outcomes .

Hypothesis 1: Primary Outcome Measure. AI/AN teens and young adults in the intervention arm will higher composite scores than participants in the control arm on physical and mental health scores from validated instruments.

Hypothesis 2: Secondary Outcome Measure. AI/AN teens and young adults who report higher composite scores in physical, mental, and spiritual health will report greater cultural resilience, identity, and cultural pride.

Hypothesis 3: Secondary Outcome Measure. AI/AN teens and young adults who report greater use of coping and mental wellness strategies will report higher composite scores in physical, mental, and spiritual health and less risky drug and alcohol misuse.

Hypothesis 4: Secondary Outcome Measure. Changes in help-seeking self-efficacy will be greatest among male participants, and young adults over 21 years old.
