# Supplementary material for: Efficacy of an mHealth Intervention (BRAVE) to Promote Mental Wellness for American Indian and Alaska Native Teenagers and Young Adults: Randomized Controlled Trial
Source: JMIR Ment Health. 2021 Sep 15;8(9):e26158. doi: 10.2196/26158 (PMC8482172; doi:10.2196/26158)
Supplement: Multimedia Appendix 3 [file mental_v8i9e26158_app3.docx]

## Multimedia Appendix 3

BRAVE Survey Questions

| Measure | Survey Questions | Answer Choices |
| --- | --- | --- |
| **Health** | 1. Rate your physical health 2. Rate your mental health 3. Rate your spiritual health | Excellent, very good, good, fair |
|  | 1. I have people to look up to 2. Getting an education is important to me 3. Friends and family know a lot about me | Strongly agree, agree, disagree, strongly disagree, don’t know/not sure, don’t want to answer |
| **Resilience** | 1. I try to finish what I start 2. I know where to go in my community to get help 3. I feel I belong at my school or work 4. My family stands by me during difficult times 5. My friends stand by me during difficult times 6. I have opportunities to develop skills that will be useful later in life | Strongly agree, agree, disagree, strongly disagree, don’t know/not sure, don’t want to answer |
| **Coping:**  **Negative** | 1. I’ve been using alcohol to make myself feel better and/or to get me through situations in life 2. I’ve been using other drugs to make myself feel better and/or to get me through situations in life | Strongly agree, agree, disagree, strongly disagree, don’t know/not sure, don’t want to answer |
| **Coping:**  **Positive** | 1. I’ve been taking action to try to make the unpleasant situation in my life better 2. I’ve been saying positive things to overcome unpleasant feelings 3. I look for something good to come out of negative things that happen to me 4. I take steps to improve my mental health and find balance 5. I have healthy outlets that I use to get through tough times | Strongly agree, agree, disagree, strongly disagree, don’t know/not sure, don’t want to answer |
| **Self-Efficacy** | 1. I am able to reach out for help when I need it 2. I am able to reach out for help when a friend needs it 3. I am able to solve problems without harming myself or others | Very confident, confident, somewhat confident, not at all confident, don’t know/not sure, don’t want to answer |
| **Self- Esteem** | 1. I am satisfied with myself 2. I have a number of good qualities 3. I have goals for the future that I work toward 4. I believe my life has purpose | Strongly agree, agree, disagree, strongly disagree, don’t know/not sure, don’t want to answer |
| **Cultural Resilience and Identity** | 1. I am proud of my ethnic background 2. I have a clear sense of my personal and cultural identity 3. I enjoy my community’s traditions 4. I feel connected to my own culture, race, or ethnic group 5. It is important for me to have indigenous values and practices 6. Spiritual beliefs are a source of strength for me | Strongly agree, agree, disagree, strongly disagree, don’t know/not sure, don’t want to answer |
| **Help Seeking** | 1. I recognize when a friend is struggling and needs my help 2. I offer help or give advice to my friends who are struggling 3. I help friends and family by getting then connected to help | Often, sometimes, rarely, never, don’t know/not sure, don’t want to answer |
