# Supplementary material for: Efficacy of an mHealth Intervention (BRAVE) to Promote Mental Wellness for American Indian and Alaska Native Teenagers and Young Adults: Randomized Controlled Trial
Source: JMIR Ment Health. 2021 Sep 15;8(9):e26158. doi: 10.2196/26158 (PMC8482172; doi:10.2196/26158)
Supplement: Multimedia Appendix 4 [file mental_v8i9e26158_app4.docx]

## Multimedia Appendix 4

**Table 8.** Treatment effect results within a subject comparing treatment to control shows no significant effects.

| Measure | n | Mean score | *P* value |
| --- | --- | --- | --- |
| **Health** | | | |
|  | 513 | -.03 | .29 |
| **Resilience** | | | |
|  | 511 | -.02 | .25 |
| **Negative coping** | | | |
|  | 468 | .03 | .41 |
| **Positive coping** | | | |
|  | 503 | -.02 | .48 |
| **Self-efficacy** | | | |
|  | 503 | -.03 | .36 |
| **Self esteem** | | | |
|  | 502 | -.05 | .07 |
| **Cultural identity** | | | |
|  | 501 | -.04 | .06 |
| **Help seeking** | | | |
|  | 502 | .07 | .09 |

Table 8 compares the treatment to control within a subject, and shows no significant findings. This analysis does not compare at specific time points. Rather, it is the within- subject analysis comparing results from BRAVE treatment period to results from STEM treatment period. For each measure and each subject, the score for that measure at the survey following BRAVE messaging was calculated and the score following STEM was calculated. Because subjects received BRAVE and STEM at different timepoints, this does not correspond to the same timepoint across subjects. For subjects who received STEM first, this compares 5 month survey – 3 month survey and for subjects who received BRAVE first this is 3 month – 5 month.

**Table 9.** Mean difference in survey measures between treatment and control groups for BRAVE and STEM intervention messaging show no significant differences.

| Measure | n | Mean treatment | Mean control | Mean difference | *P* value |
| --- | --- | --- | --- | --- | --- |
| **Health** | | | | | |
|  | 616 | .07 | .05 | .02 | .71 |
| **Resilience** | | | | | |
|  | 613 | .03 | .02 | .01 | .76 |
| **Negative coping** | | | | | |
|  | 541 | .07 | .000 | .07 | .35 |
| **Positive coping** | | | | | |
|  | 603 | .07 | .05 | .03 | .59 |
| **Self-efficacy** | | | | | |
|  | 599 | .12 | .11 | .002 | .98 |
| **Self esteem** | | | | | |
|  | 595 | .095 | .09 | .007 | .89 |
| **Cultural identity** | | | | | |
|  | 599 | .006 | -.03 | .04 | .33 |
| **Help seeking** | | | | | |
|  | 597 | .16 | .09 | .07 | .36 |

Table 9 shows the mean difference in scores between treatment and control groups for survey messaging. The mean difference in a score is the difference between treatment and control for a particular survey measure. In this study, changes cannot be tracked within a subject since subjects have not received both STEM and BRAVE messages.
