# Supplementary material for: Efficacy of an mHealth Intervention (BRAVE) to Promote Mental Wellness for American Indian and Alaska Native Teenagers and Young Adults: Randomized Controlled Trial
Source: JMIR Ment Health. 2021 Sep 15;8(9):e26158. doi: 10.2196/26158 (PMC8482172; doi:10.2196/26158)
Supplement: Multimedia Appendix 5 [file mental_v8i9e26158_app5.pdf]

Effectiveness of an mHealth Intervention (BRAVE) to Promote Mental Wellness for American Indian and Alaska Native Teenagers and Young Adults: Randomized Controlled Trial

BRAVE

Methods

Culturally relevant interventions are needed to help American Indian and Alaska Native (AI/AN) teenagers and young adults.

- In 2019, 2334, AI/AN teenagers and young adults nationwide recruited via social media channels and SMS text messages, enrolled 1044 participants.
- Participants received either BRAVE SMS text messages or 8 weeks of science, technology, engineering, and math (STEM) SMS text messages.
- Impacts tested using linear mixed-effect models and linear regressions.

Findings

833 AI/AN teenagers and young adults in the BRAVE and STEM arms showed significant positive trends for most measured outcomes, except cultural identity and help-seeking behavior.

79% of participants used SMS text messages to help themselves.

54% of participants used SMS text messages to help friends or family members.

Statistically significant increases in **health, resilience, positive coping, self-efficacy, and self-esteem** scores (P<.005).

Conclusion

This is the first national randomized controlled trial of a mobile health intervention among AI/AN teenagers and young adults to test the efficacy of a mental wellness intervention in relation to STEM career messages. This study provides new insights for supporting the next generation of AI/AN changemakers.

Text

Native youth and young adults can now **text BRAVE to 97779** or **STEM to 97779** to receive the SMS text message sequence on their own.

Reference

Craig Rushing et.al. Effectiveness of an mHealth Intervention (BRAVE) to Promote Mental Wellness for American Indian and Alaska Native Teenagers and Young Adults: Randomized Controlled Trial. JMIR Mental Health, 2021.
